# A cross-sectional study of adolescent non-suicidal self-injury: support for a specific distress-function relationship

**DOI:** 10.1186/1753-2000-8-23

**Published:** 2014-08-08

**Authors:** Maria Zetterqvist, Lars-Gunnar Lundh, Carl Göran Svedin

**Affiliations:** 1 Department of Clinical and Experimental Medicine; Child and Adolescent Psychiatry, Linköping University, Linköping SE-581 85, Sweden; 2 Child- and Adolescent Psychiatric Clinic, University Hospital, Linköping SE-581 85, Sweden; 3 Department of Psychology, Lund University, Lund SE-221 00, Sweden

**Keywords:** Non-suicidal self-injury, Adolescents, Function, Adverse life events, Trauma symptoms

## Abstract

**Background:**

This study has investigated the specific relationship between childhood adversities, individual trauma symptoms and the functions of non-suicidal self-injury (NSSI). The aim was to examine whether different self-reported adverse experiences and trauma symptoms predict the need to engage in NSSI, either to regulate emotions or to communicate with and influence others.

**Method:**

The participants were a community sample of 816 adolescents aged 15–17 years with NSSI. Hierarchical multiple regression was used, controlling for NSSI frequency and gender. The dependent variables were the automatic and social functions of NSSI, respectively. The predictors entered in the model were several different maltreatment and adversity experiences as well as individual trauma symptoms. Mediation analyses were also performed using the bootstrapping method with bias-corrected confidence estimates.

**Results:**

Frequency of NSSI, gender (female), emotional abuse, prolonged illness or handicap during upbringing and symptoms of depression uniquely predicted the automatic functions of NSSI in the final regression model, but not the social functions. Symptoms of anxiety uniquely predicted social but not automatic functions. Having experienced physical abuse, having made a suicide attempt and symptoms of dissociation were significant predictors in both final models. The model for automatic functions explained more of the variance (62%) than the social model (28%). The relationship between childhood emotional, physical and sexual abuse and performing NSSI for automatic reasons was mediated by symptoms of depression and dissociation. The relationship between physical abuse and the social functions of NSSI was mediated by symptoms of anxiety and dissociation.

**Conclusions:**

It is important to understand the specific context in which NSSI has developed and is maintained. Experiences of emotional abuse and symptoms of depression could guide clinical work in the direction of emotion regulation skills since in this study these variables were uniquely associated with the need to engage in NSSI to regulate emotions, to self-punish or to generate feelings. The presence of physical abuse, a suicide attempt and symptoms of dissociation could alert clinicians to a broad treatment approach since they were associated with performing NSSI to regulate both social and automatic experiences.

## Background

### Non-suicidal self-injury and childhood adversities

Non-suicidal self-injury (NSSI), defined as the deliberate destruction of body tissue without suicidal intent [1], is a prevalent condition during adolescence [2,3]. In order to identify adolescents at risk and to develop tailored interventions, the mechanisms behind NSSI and the context in which NSSI emerges and is maintained have become the subject of growing interest. In this context, the relationship between child maltreatment (such as sexual, physical or emotional abuse) and NSSI has been examined (e.g., [4,5]), but with some inconsistent results. While some researchers, for example, have found support for a relationship between physical abuse and NSSI (e.g., [5-9]), others have not [4,10]. Similarly, results have also differed concerning sexual abuse and NSSI, where an association has been found in some studies [11-13], while Klonsky and Moyer [14] in their meta-analysis showed that the relationship between sexual abuse and NSSI is in fact relatively small. Despite these inconsistencies, there is general agreement that childhood maltreatment is one of several factors to be considered along the pathway leading to NSSI. However, the specific association between maltreatment experience and NSSI has turned out to be complex, suggesting that the relationship between maltreatment and negative health outcomes is also associated with the same risk factors, such as high risk family environments, or different mediators [14]. Another more recent review [15] also reached the same conclusion and pointed out that although sexual abuse is a significant risk factor for both suicidal and non-suicidal self-injury, it should be considered general and non-specific, and ideally other potentially confounding biological, psychological and social risk factors should be controlled for when analyzing the relationship. In other words, it is not necessarily the abuse on its own, but also the quality of the family context in which it occurs, that contributes to NSSI [4].

There is empirical support for the effect of invalidating family environments on NSSI, such as family relational problems, criticism, fear and alienation in the parent–child relationship as well as perceived lack of family support (e.g., [6,16-18]). High parental expressed emotion, in particular parental criticism, has been found to be associated with NSSI in adolescents [19]. Further support for this has been found in longitudinal studies where family invalidation predicted the occurrence of NSSI in adolescents [20]. Thus, findings suggest that it is not only the experience of direct maltreatment that contributes to NSSI but also the quality of the family environment. In addition to environmental factors, individual psychopathology also needs to be taken into account in the conceptualization of how NSSI emerges and is maintained. In a sample of Turkish high school students, Zoroglu et al. [9] found that trauma and dissociation contributed to self-mutilation, with dissociation being especially evident. In a population sample of 4019 adolescents, Tolmunen et al. [21] showed that high levels of dissociation were independently associated with current self-cutting. In a large community sample of adolescents, Zetterqvist et al. [22] also found more self-reported experience of adversities and trauma symptoms such as depression, dissociation and posttraumatic stress in adolescents with more frequent NSSI, compared to those with only occasional NSSI. Depressive symptoms have predicted NSSI in several longitudinal adolescent samples [20,23-25].

During recent years, it has been suggested that individual factors such as these mediate the relationship between maltreatment and self-injury. By examining proximal mediating factors, our understanding of the pathways that underlie the development of NSSI in adolescents and young adults has been expanded. A number of these different factors have been found to mediate the relationship between maltreatment and self-injury, such as alexithymia [7,26], posttraumatic stress [27], especially in relation to sexual abuse [28], and dissociation [7] also, again, in connection with sexual abuse in particular [10,12,29]. Furthermore, self-criticism statistically mediated the relationship between emotional abuse during childhood and engagement in NSSI during adolescence [11]. Gratz and Roemer [30] have also stressed the importance of emotion regulation skill deficiencies in understanding NSSI. Early maltreatment and less optimal upbringing experiences are thought to influence the capacity for emotion regulation and communication skills, increasing the need for NSSI as a coping behavior. Thus, the mechanisms whereby environmental factors, such as child maltreatment, are related to NSSI can be better understood by also examining the proximal mediating effect of individual psychopathology.

### A specific distress-function relationship

It is important to understand the context in which the need to use NSSI to regulate emotional and social experiences is developed and maintained. It is generally thought that invalidating and insensitive caregiving environments have a detrimental effect on children’s development, rendering them vulnerable and making it difficult for them to reflect on affective experiences, for example, or to use language to describe and share inner states with others. In this context NSSI can function as a compensatory regulatory strategy [12,31]. During recent years, research has moved beyond general descriptions of NSSI functions and begun to examine more specific relationships between psychosocial variables and the functions of NSSI, lending support to the validity of the function model of automatic/intrapersonal and social/interpersonal functions of NSSI. Automatic functions refer mainly to emotion regulation, tension reduction, feeling-generation and self-punishment. Social/interpersonal functions refer to performing NSSI to communicate with and influence others, as well as identification with peers and the avoidance of social demands ([32,33] unpublished observations). According to prior research, engaging in NSSI for automatic/intrapersonal reasons has most clearly been associated with symptoms of depression, posttraumatic stress and dissociation [10,32,34-36], self-criticism [11], sexual abuse [10,35,37], emotional abuse [10], thought and expressive suppression [38,39], physiological arousal [40], suicide ideation [32] and suicide attempts [36], whereas social functions have been associated with interpersonal distress [34], social perfectionism and social concerns [36] and paternal antipathy [37], and negatively associated with expressive suppression [39]. Recurrent NSSI has been found to be associated with intrapersonal motives for self-injurious behaviors [12]. Turner et al. [39] similarly found an association between NSSI lifetime frequency and automatic/intrapersonal functions, but not interpersonal functions, and Klonsky and Olino [41] found one group of severe self-injurers that mainly reported automatic functions. Other studies have found that more severe NSSI and higher scores on clinical measures have been related to more overall reported functions, both social and automatic [2,32]. NSSI frequency has evoked recent interest, due to the inclusion of NSSI in section III of the fifth version of the Diagnostic and Statistical Manual of Mental Disorders (DSM-5) [42], with criterion A suggesting clinical significance at a prevalence rate of 5 or more days of NSSI during the last year.

Examining function-specific correlates contributes knowledge of possible pathways as to how environmental adversities and individual factors may influence an individual to engage in NSSI to achieve specific goals, such as emotion regulation and/or influencing others, taking us an important step further toward developing functionally relevant interventions [43] for NSSI. The current study aims to examine the mechanisms that are associated with engaging in NSSI for either social or automatic purposes. Due to the large sample of self-injuring adolescents, we can explore the specific relationship between several self-reported adverse experiences during childhood and different individual trauma symptoms and the functions of NSSI. Based on previous studies, we predicted that there would be specific relationships between adverse life events and symptoms of traumatic stress and the automatic and social functions, respectively, and that the relationship between adverse life events and specific functions of NSSI is mediated by trauma symptoms. More specifically, we predicted that maltreatment, such as emotional, physical and sexual abuse, would be significant predictors for automatic functions, performing NSSI for reasons of emotional regulation or self-punishment. A suicide attempt would also be associated with automatic functions. We proposed that trauma symptoms, specifically symptoms of depression, dissociation and posttraumatic stress, would predict automatic functions of NSSI in the multivariate model and also mediate the relationship between childhood maltreatment and the automatic/intrapersonal functions of NSSI. Relatively less research has been carried out on the correlates of social functions, and previously proposed models have resulted in a relatively low explained variance (e.g., [39]). Similarly to Hilt et al. [34], we predicted that experiences of bullying would be associated with social functions, but apart from this we chose not to specify the relationship exactly and thus analyses of the social functions were explorative.

## Methods

### Participants

The participants were 816 adolescents aged 15–17 years who confirmed at least one episode of NSSI during the past year, and who were taken from a randomized community sample of 3097 adolescents from the county of Östergötland in the south east of Sweden. For more information on the larger sample, see Zetterqvist et al. [3]. Of the 1088 adolescent who confirmed at least one episode of NSSI during the past year, 836 responded to all items in the Functional Assessment of Self-Mutilation (FASM) about the functions of their self-injury and were included for further analyses. An additional 20 adolescents were excluded due to missing data on questionnaires Linköping Youth Life Experience Scale (LYLES) and/or Trauma Symptom Checklist for Children (TSCC). This resulted in 816 individuals for further analysis.

The 272 excluded adolescents did not differ from those included on background demographics such as gender, parents’ or own country of origin, education, living conditions and parents’ occupational status. However, there were significant differences on self-injury status, with fewer adolescents in the excluded group having made a suicide attempt. The excluded group also reported less frequent NSSI, as well as less reported moderate/severe NSSI as defined by Lloyd et al. [44] The excluded adolescents also reported lower levels of trauma symptoms and number of interpersonal and chronic adversities.

### Procedure

The headmaster/headmistress of each school was given information about the study and gave their consent for the school to participate. One week prior to our visit in the classroom, teachers distributed written information about the study. Students and parents were informed that participation was voluntary, and if the students wished to participate in the study they should show up in class the following week when the data collection would take place. According to the Ethical Review Act [45] of Sweden, active consent is not required from parents when adolescents are 15 years of age or older. Parents were informed that they were welcome to contact the research group if they had any questions. Data collection was performed in the classroom, with desks placed sufficiently far apart to ensure anonymity. The questionnaires consisted of twelve pages and took approximately 25–30 minutes to complete. The participants and procedure have been described in detail in a previous study [3].

### Ethical issues

The study was approved by the Regional Ethical Review Board of Linköping (Dnr, 2010/195-31). During the data collection, students were encouraged to seek professional help if needed and given written contact information to several local counseling alternatives.

### Measures

#### Non-suicidal self-injury

The Functional Assessment of Self-Mutilation (FASM; [44]) assesses the methods, frequency and function of self-reported deliberate NSSI. Respondents are asked whether they have engaged in any of eleven different forms of NSSI during the past year or at any time previously. The frequency of NSSI is also assessed. FASM contains 22 statements assessing the functions of NSSI, which respondents rate on a four-point Likert scale, ranging from “never” to “often”. FASM has previously been used in normative [44] and psychiatric samples [46]. FASM has shown acceptable psychometric properties in adolescent samples [46,47], with adequate internal consistency for both minor and moderate/severe forms of NSSI. There is also support for the concurrent validity of FASM demonstrating significant associations with measures of recent suicide attempt, hopelessness and depressive symptoms [36]. The Swedish version of FASM was translated into Swedish using a back-translation procedure and tested in a pilot study. The psychometric properties of the Swedish version have been fully described in another article by Zetterqvist et al. [3]. Cronbach’s alpha for the present sample on all NSSI items was *α* = .80. Results for the subscales referred to as minor and moderate/severe forms of NSSI [44] was *α* = .64 and .70, respectively. Alpha for the FASM functions of both automatic/intrapersonal and social/interpersonal for the present sample was: α = .86. Based on learning theory, Nock and Prinstein [33] have previously confirmed a four-factor model of FASM functions, with an underlying factor structure of automatic negative, automatic positive, social negative, and social positive reinforcement. The two factors automatic and social functions used as dependent variables in the analysis in this study are based on results from previous factor analysis on the present sample of Swedish community adolescents ([3] unpublished observations).

#### Suicidal self-injury

The presence of a suicide attempt was assessed with the suicide intent question from FASM and the question: “Have you ever made an actual attempt to kill yourself in which you had at least some intent to die?” (Yes/No), from the Self-Injurious Thoughts and Behavior Interview Short-Form Self-Report (SITBI-SF-SR), which was developed from SITBI [48]. For translation and psychometric data on Swedish adolescents, see [3]. Two additional questions were also developed for the purpose of this study (“Have you ever intentionally taken an overdose of medicine or swallowed other substances with the intention of hurting yourself?” and “If so, was it your intention to kill yourself when you performed the act?”).

#### Potentially traumatic life events and adversities

Linköping Youth Life Experience Scale (LYLES) is an instrument for gauging potentially traumatic life events, including adverse childhood circumstances. It has been developed from Life Incidence of Traumatic Experiences [49]. LYLES contains 23 main questions with more detailed secondary items; 18 items are considered non-interpersonal (such as being in a car accident, staying in hospital), 13 items interpersonal (such as having been exposed to physical or sexual abuse or threatened), and 10 items ask questions about more longstanding adverse childhood circumstances (such as parental alcohol abuse, parent in jail). LYLES is intended to cover several important types of potentially traumatic events and circumstances during an adolescent’s lifespan. LYLES has been evaluated on Swedish adolescents from the normative population. Its psychometric properties have been shown to be satisfactory with test-retest *r* = .79 and kappa item per item ranging between *k* = .44 and 1.0 [50]. In the present study, only the items assessing direct experience of maltreatment and adversities from the interpersonal and adverse circumstances subscales were used (response yes/no). A combination variable, “parental chronic adversity” was created post hoc by adding items “separated from parents”, “parental divorce”, “parental quarreling after divorce”, “parental drug or alcohol problem”, “parental mental health problems”, “prolonged illness or handicap”, and “parent in jail”. Bullying was assessed with a single item from LYLES: “Have you ever been exposed to bullying?” (Yes/No).

#### Trauma symptoms

The Trauma Symptom Checklist for Children (TSCC; [51]) is a self-report questionnaire developed to identify symptoms of traumatic stress in children and adolescents aged 8–17 years. The questionnaire consists of 54 items and the respondents rate their answers on a four-point Likert scale from 0 (never) to 3 (almost always). The results are divided into six subscales: anxiety, depression, anger, posttraumatic stress, sexual preoccupation and dissociation, with 9–10 items in each. TSCC has been translated into Swedish and evaluated on Swedish children and adolescents [52]. Good reliability such as internal consistency (Cronbach’s alpha) for the total scale .94 (ranging in the clinical scales .78-.83) and test-retest for the total scale *r* = .81 (ranging in the clinical scales .67-.81) has been found. The confirmatory 6-factor analysis explained 50.7% of the variance. Other validity measures, such as concurrent validity and criterion-related validity, also were shown to be satisfactory. The subscale sexual concern was not used in this study. Internal consistency was good for the subscales used in the present sample, *α* = .90 (depression), *α* = .84 (anxiety), *α* = .85 (anger), *α* = .90 (posttraumatic stress), *α* = .87 (dissociation). In accordance with the TSCC manual [51], individuals with six or more missing items on the total scale and three or more missing on each subscale were excluded from analyses. Single missing items were replaced with the average value on that subscale.

#### Demographic information

A demographic questionnaire was created for the purpose of the study, assessing demographic characteristics such as gender, type of education, own and parents’ country of origin, perception of family’s economy, living conditions and parents’ occupation. Adolescents self-reported demographic information in fixed answer categories.

### Data analysis

Categorical data were analyzed with descriptive statistics using frequencies and cross-tabulation with Chi square. Phi coefficient was calculated for effect size (ES). Multiple hierarchical linear regression analysis was used, controlling for NSSI frequency and gender. The dependent variables were the social and automatic functions of NSSI, respectively. The additional predictors/explaining variables entered into the model were different self-reported maltreatment/adverse childhood experiences and trauma symptoms (depression, posttraumatic stress, anxiety, anger, dissociation), as well as having made a suicide attempt. There was violation of the assumption of homoscedasticity for the social functions and the standard errors were therefore adjusted, using the heteroscedasticity-consistent standard error estimator [53]. Mediation was tested using the bootstrapping method with bias-corrected confidence estimates [54] and the 95% CI of the indirect effects was obtained with 5000 bootstrapping resamples [55]. All statistical analyses were performed using the SPSS 20.0 software package with macros (HCREG and INDIRECT) downloaded from Hayes [56].

## Results

Of the 816 adolescents who had engaged in NSSI during the past year, 287 (35.2%) reported NSSI 1–4 times, 165 (20.2%) reported 5–10 times and the remaining 364 (44.6%) adolescents reported NSSI ≥ 11 times. Regarding type of NSSI reported in the sample, 577 (70.7%) reported at least one episode of NSSI that Lloyd et al. [44] refer to as “moderate/severe”, which includes cutting/carving, burning, self-tattooing, scraping and erasing skin. Of these, 270 (46.8%) reported ≥ 5 incidents of these types of NSSI. Of the 816 adolescents with NSSI, 137 (16.9%, *N* = 811) also reported a life-time prevalence of suicide attempts. Mean and standard deviation for the automatic and social functions reported was 4.51 (4.88) and 2.75 (4.71), respectively. Mean and standard deviation for the traumatic symptoms was for depression: 6.87 (5.70), anxiety: 5.88 (4.73), anger: 6.24 (5.04), posttraumatic stress: 9.17 (6.49) and dissociation: 7.93 (5.92). Sociodemographics, health-related variables and frequencies of interpersonal maltreatment and chronic adversities for the whole sample and also boys and girls separately are presented in Table 1.

**Table 1:** Frequencies and percentages regarding demographics, health-related variables and self-reported experience of interpersonal maltreatment and chronic adversity in a sample of adolescents with NSSI

**Variables**	**All**	**Girls**	**Boys**
	***n*** **= 771-816**	***n*** **= 437-465**^**x**^	***n*** **= 331-348**^**x**^
Demographic variables			
Born in Sweden	746 (92.0)	433 (93.3)	310 (90.1)
Parents’ born in Sweden	607 (74.5)	355 (76.3)	250 (72.0)
Theoretical education	357 (43.8)	213 (45.8)	143 (41.1)
Both parents working	572 (74.2)	324 (74.1)	247 (74.6)
Living with both parents^†^	554 (68.2)	309 (66.5)	244 (70.9)
Health-related variables			
Ever smoked	461 (57.1)	276 (59.6)	183 (53.5)
Ever tried alcohol	648 (79.6)	369 (79.4)	276 (79.8)
Ever used drugs	129 (15.9)	64 (13.8)	64 (18.4)
Has friend who has self-injured	560 (69.3)	379 (82.4)	179 (51.9)
Ever had professional contact*	378 (46.8)	250 (54.5)	125 (36.2)
Experience of interpersonal maltreatment and chronic adversity			
Bullied	368 (45.1)	233 (50.1)	132 (37.9)
Prolonged illness or handicap	111 (13.6)	63 (13.5)	48 (13.8)
Emotional abuse	401 (49.1)	274 (58.9)	124 (35.6)
Physical abuse by adult in family	173 (21.2)	116 (24.9)	56 (16.1)
Sexual abuse by adult in family or other	121 (14.8)	110 (23.7)	10 (2.9)
Parental chronic adversity^+^	466 (57.1)	292 (62.8)	171 (49.1)

*Note.* NSSI = non-suicidal self-injury, ^x^there are three individuals with missing data on gender, which explains why the number of girls and boys do not add up to the total, ^†^together or every- other week, *not specifically for self-injury, ^+^including alcohol or drug abuse, mental health problems, in jail, prolonged handicap or illness, separated from parent(s), divorce, quarreling during divorce.

Of the sample of 816 self-injuring adolescents, 186 (22.8%) adolescents fulfilled all the NSSI diagnostic criteria (see a previous study by Zetterqvist et al. [3] for information on how DSM-5 NSSI criteria were operationalized and applied empirically). To elaborate further on NSSI frequency and NSSI disorder and the relationship to maltreatment and adverse life events, the 186 adolescents with NSSI disorder were compared to the 630 adolescents that did not fulfill DSM criteria. Significantly more adolescents (*p* < .001) among those fulfilling NSSI criteria reported having experienced bullying (116 [62.4%] vs. 252 [40.0%]), emotional abuse (144 [77.4%] vs. 257 [40.8%]), physical abuse (72 [38.7%] vs. 101 [16.0%]), sexual abuse (68 [36.6%] vs. 53 [8.4%]) as well as parental chronic adversity (129 [69.4%] vs. 337 [53.5%]) during their lifetime. The difference for emotional and sexual abuse reached medium ES (phi = .31 and .33, respectively), whereas the ES for the other maltreatment and adversities variables was small. Furthermore, adolescents meeting diagnostic NSSI criteria reported significantly higher levels of trauma symptoms (*p* < .001) on the subscales of TSCC (depression, anxiety, anger, posttraumatic stress and dissociation).

### Multiple regression analyses

Given probable overlap among different maltreatment experiences and trauma symptoms, the variables were included together in a multivariate model to examine unique prediction for either automatic/intrapersonal or social/interpersonal functions. Gender and frequency of NSSI have previously been shown to influence the functions of NSSI [3,12,32,39] and were controlled for in the analysis, in line with a previous study by Turner et al. [39]. All the included maltreatment and adversity items from LYLES and trauma symptom subscales from TSCC were significant in the zero-order correlation analysis (Table 2). In the hierarchical multiple regression model of the automatic functions of NSSI, NSSI frequency was entered as step 1, explaining 24% of the variance in the automatic functions. Gender, entered as step 2, explained an additional 12%. After controlling for frequency and gender, the maltreatment and adversities items entered in step 3 explained an additional 13%, *R* squared change = .13, *F* change (7, 798) = 28.11, *p* < .001. The trauma symptoms entered next explained an additional 13%, *R* squared change = .13, *F* change (5, 793) = 52.56, *p* < .001. The total variance explained by the model as a whole was 62%, *F* (14, 793) = 91.28, *p* < .001. In the final model, NSSI frequency, gender (female), having made a suicide attempt, being exposed to physical abuse by an adult in the family, emotional abuse, having a long-term handicap or illness during upbringing and symptoms of depression and dissociation were significant (see Table 3). When frequency of NSSI was not included in the analysis, there was a non-significant trend for sexual abuse in the final model (*p* = .067).

**Table 2:** Correlations of self-reported experience of childhood adversities and symptoms of traumatic stress with automatic and social functions of NSSI

**Variables**	**Automatic functions**	**Social functions**
Frequency of NSSI	.49***	.31***
Gender	.39***	.18***
Suicide attempt	.47***	.32***
Adverse Childhood Circumstances (ACC) during upbringing		
Bullied	.29***	.22***
Prolonged illness or handicap	.12***	.12***
Parental chronic adversity†	.20***	.11**
Emotional abuse	.41***	.26***
Interpersonal Negative Events (IPE)		
Physical abuse by adult in family	.29***	.28***
Sexual abuse by adult in family or other	.35***	.25***
Symptoms of traumatic stress		
Depression	.73***	.43***
Anxiety	.58***	.42***
Anger	.44***	.34***
Posttraumatic stress	.62***	.42***
Dissociation	.62***	.43***

*Note.* NSSI = non-suicidal self-injury, *n* = 808 in the model of social functions, due to listwise deletion of missing data being default option in the heteroscedasticity-consistent standard error (HCSE) macro [53], † including alcohol abuse, mental health problems, in jail, handicap, separated from parent(s), divorce, quarreling during divorce, **p <.01, ***p <.001.

**Table 3:** Hierarchical multiple regression for the automatic and social functions of NSSI

	**Automatic functions**						**Social functions**					
**Block of predictors**	***R***^***2 ***^ ***change***	***B***	***SE B***	***β***	***t***	***p***	***R***^***2 ***^ ***change***	***B***	***SE (HC)***^**†**^	***β***	***t***^**†**^	***p***^**†**^
1 Frequency of NSSI	.24***	.05	.01	.18	6.79	**<.001**	.09***	.02	.01	.07	1.42	.155
2. Gender (female)	.12***	.95	.27	.10	3.53	**<.001**	.03***	−.21	.42	−.02	−.50	.617
3. Self-reported exposure to adversities	.13***						.10***					
Bullied		−.36	.26	−.04	−1.42	.157		.25	.35	.03	.70	.483
Having made a suicide attempt		1.78	.34	.14	5.29	<.001		1.57	.63	.12	2.51	.012
Physical abuse by adult in family		.57	.29	.05	1.99	.047		1.42	.53	.12	2.68	.008
Sexual abuse by adult in family or other		.39	.34	.03	1.14	.254		.77	.56	.06	1.37	.170
Emotional abuse		.61	.26	.06	2.31	**.021**		.12	.37	.01	.31	.755
Parental chronic adversity		.15	.23	.02	.65	.518		−.21	.31	−.02	−.67	.505
Prolonged illness or handicap during upbringing		.75	.32	.05	2.34	**.020**		.79	.51	.06	1.55	.121
4. Self-reported symptoms of traumatic stress	.13***						.06***					
Depression		.34	.04	.40	9.37	**<.001**		.03	.06	.03	.49	.622
Anxiety		.004	.04	.004	.09	.927		.17	.07	.16	2.45	**.014**
Anger		−.04	.03	−.04	−1.35	.178		.03	.04	.04	.79	.427
Posttraumatic stress		.02	.03	.03	.66	.512		−.001	.04	−.002	−.03	.975
Dissociation		.11	.03	.14	3.61	<.001		.12	.05	.15	2.48	.013

*Note.* NSSI = non-suicidal self-injury, boldface values represent unique relationship between predictor and automatic or social functions, all statistics (except *R*^2^ change) presented in the table refer to the final fourth model, ^†^values adjusted using the heteroscedasticity-consistent standard error (HCSE) estimator [53], *n* = 808 in the model of social functions, due to listwise deletion of missing being the default option in the HCSE macro, ***p < .001.

In the hierarchical multiple regression model of the social functions of NSSI, NSSI frequency was entered as step 1, explaining 9% of the variance in the social functions. Gender, entered as step 2, explained an additional 3%. After controlling for frequency and gender, the maltreatment and adversities items entered in step 3 explained an additional 10%, *R* squared change = .10, *F* change (7, 798) = 13.75, *p* < .001. The trauma symptoms entered next explained an additional 6%, *R* squared change = .06, *F* change (5, 793) = 13.58, *p* < .001. The total variance explained by the model as a whole was 28%, *F* (14, 793) = 21.65, *p* < .001. After adjusting the standard errors using the heteroscedasticity-consistent standard error estimator in the final model of social functions (*R* square = .28, *F* (14, 793) = 14.37, *p* < .001), having made a suicide attempt, being exposed to physical abuse by an adult in family and symptoms of anxiety and dissociation were significant (see Table 3).

NSSI frequency, gender (female), emotional abuse, long-term illness or handicap during upbringing and symptoms of depression uniquely predicted automatic functions in the final regression model, and were not significant predictors of the social functions. Symptoms of anxiety uniquely predicted social but not automatic functions. Having made a suicide attempt, being exposed to physical abuse and symptoms of dissociation were significant predictors in both models (Table 3). The model for automatic functions explained more of the variance than did the predictors for social functions (62% vs 28%). There was a non-significant trend for sexual abuse as a unique predictor of automatic functions when NSSI frequency was not included in the model. We therefore further examined whether NSSI frequency mediated the relationship between sexual abuse and automatic functions. Results showed that NSSI frequency was a significant mediator (CI = 1.00-2.19) of the relationship.

### Mediation analyses

The maltreatment events that were significant in the final regression model for the automatic functions (emotional and physical abuse) and social functions (physical abuse) respectively were entered into a mediation analysis. Mediation for sexual abuse and automatic functions was also tested. The trauma symptoms that were significant in the same model were entered as mediators to illustrate the relationship between environmental maltreatment and individual symptoms and the functions of NSSI. It is preferable to test several theoretically relevant mediators between maltreatment and NSSI simultaneously in a multiple mediation model because the mediators often co-occur [27]. We therefore conducted multiple regression analyses to assess each component of the proposed mediation models. The significant mediators in the two regression models did in fact correlate (symptoms of depression-dissociation *r* = *.*74, symptoms of anxiety-dissociation *r =* .68), but there was no multi-collinearity in the regression models.

#### Automatic functions of NSSI

**Figure 1:**
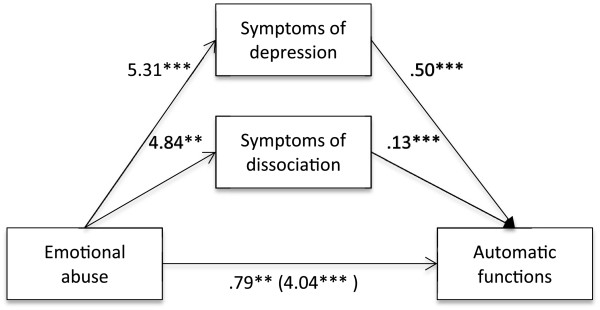
**Indirect effect of emotional abuse on automatic functions through symptoms of depression and dissociation.***Note*. **p <. 01. ***p <. 001.

**Figure 2:**
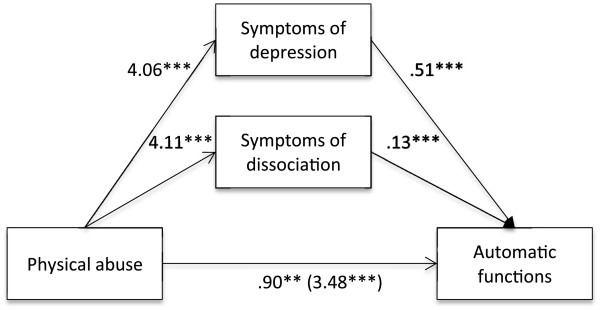
**Indirect effect of physical abuse on automatic functions through symptoms of depression and dissociation.***Note*. **p <. 01. ***p <. 001.

**Figure 3:**
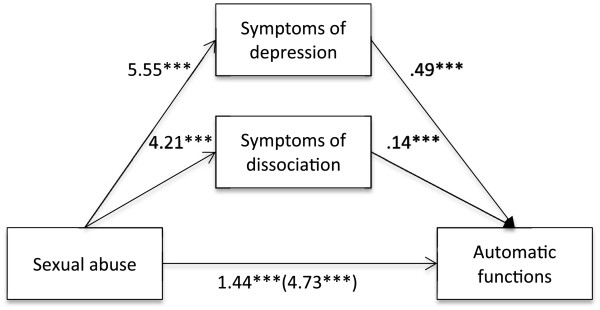
**Indirect effect of sexual abuse on automatic functions through symptoms of depression and dissociation.***Note.* ***p <. 001.

Firstly, it was found that self-reported experience of emotional (B = 4.04, *t* (813) = 12.97, *p* < .001), physical (B = 3.48, *t* (813) = 8.72, *p* < .001) and sexual abuse (B = 4.73, *t* (813) = 10.48, *p* < .001), separately, were positively associated with the automatic functions of NSSI. It was also found that self-reported experience of emotional, physical and sexual abuse, separately, was positively associated with symptoms of depression (emotional abuse: B = 5.31, *t* (813) = 15.01, *p* < .001; physical abuse: B = 4.06, *t* (813) = 8.69, *p* < .001; sexual abuse: B = 5.55, *t* (813) = 10.53, *p* < .001) as well as dissociation (emotional abuse: B = 4.84, *t* (813) = 12.80, *p* < .001; physical abuse: B = 4.11, *t* (813) = 8.45, *p* < .001; sexual abuse: B = 4.21, *t* (813) = 7.45, *p* < .001). Lastly, it was found that the mediators, symptoms of depression (emotional abuse: B = .50, *t* (813) = 15.93, *p* < .001; physical abuse: B = .51, *t* (813) = 16.84, *p* < .001; sexual abuse: B = .49, *t* (813) = 15.88, *p* < .001) and dissociation (emotional abuse: B = .13, *t* (813) = 4.39, *p* < .001; physical abuse: B = .13, *t* (813) = 4.39, *p* < .001; sexual abuse: B = .14, *t* (813) = 4.80, *p* < .001), were associated with the automatic functions of NSSI (Figures 1, 2 and 3). Because both the a-paths and the b-paths were significant in all three models, mediation analyses were tested using the bootstrapping method with bias-corrected confidence estimates [54]. In the present study, the 95% CI of the indirect effects was obtained with 5000 bootstrapping resamples [55]. Results of the mediation analyses confirmed the mediating role of trauma symptoms (depression and dissociation) in the relationship between self-reported experiences of emotional abuse (B = 3.24, CI = 2.79-3.73), physical abuse (B = 2.58, CI = 1.99-3.18), as well as sexual abuse (B = 3.28, CI = 2.60-4.04) and the automatic functions of NSSI in all three models. The results indicated that the direct effect of emotional abuse (B = .79, *t* (813) = 3.05, *p* = .002) and physical abuse (B = .90, *t* (813) = 3.05, *p* = .002) as well as sexual abuse (B = 1.44, *t* (813) = 4.22, *p* < .001) on the automatic functions of NSSI, when controlling for symptoms of depression and dissociation, was still significant (Figures 1, 2 and 3).

#### Social functions of NSSI

**Figure 4:**
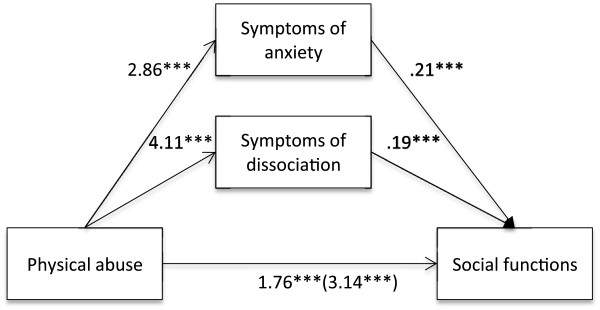
**Indirect effect of physical abuse on social functions through symptoms of anxiety and dissociation.***Note.* ***p <. 001.

Firstly, it was found that self-reported experience of physical abuse was positively associated with the social functions of NSSI (B = 3.14, *t* (813) = 8.07, *p* < .001). It was also found that self-reported experience of physical abuse was positively associated with symptoms of anxiety (B = 2.86, *t* (813) = 7.28, *p* < .001) as well as dissociation (B = 4.11, *t* (813) = 8.45, *p* < .001). Lastly, it was found that the mediators, symptoms of anxiety (B = .21, *t* (813) = 4.97, *p* < .001) and dissociation (B = .19, *t* (813) = 5.59, *p* < .001), were associated with the social functions of NSSI. Results of the mediation analyses confirmed the mediating role of symptoms of anxiety and dissociation in the relationship between self-reported experiences of physical abuse (B = 1.38, CI = .98-1.84) and the social functions of NSSI. The results indicated that the direct effect of physical abuse (B = 1.76, *t* (813) = 4.73, *p* < .001) on the social functions of NSSI, when controlling for symptoms of anxiety and dissociation, was still significant (Figure 4).

## Discussion

In this sample of self-injuring adolescents, significantly more adolescents who fulfilled diagnostic criteria for NSSI reported having experienced emotional, physical and sexual abuse and reported higher levels of trauma symptoms than did adolescents with NSSI who did not fulfil diagnostic criteria. These results can potentially explain why adolescents with NSSI disorder experience negative feelings/thoughts or interpersonal difficulties (DSM-5 criterion C), and thus feel the need to engage in repetitive NSSI (DSM-5 criterion A) in order to relieve negative feelings, cognitive states, interpersonal difficulties or to induce positive feelings (DSM-5 criterion B), as this regulatory function reinforces the behavior. This cross-sectional study investigated the specific relationship between childhood adversities, individual trauma symptoms and the functions of NSSI. Due to the large sample, several predictors were included in the multiple regression models, investigating the role of both environmental and individual factors in automatic and/or social functions of NSSI. The present study found support for the specificity of a distress-function relationship, with NSSI frequency, gender, emotional abuse, prolonged illness or handicap and symptoms of depression uniquely predicting automatic but not social functions, and symptoms of anxiety uniquely predicting social but not automatic functions of NSSI.

### Automatic functions of NSSI

Frequency of NSSI was a significant predictor in the automatic model but not for the social functions. This implies that more frequent NSSI is associated with the need to engage in NSSI to regulate emotions, punish oneself or to generate feelings. There is some support for this in Klonsky and Olino’s [41] study where one group of severe self-injurers mainly reported automatic functions, as well as a study by Turner et al. [39], who found that NSSI frequency was associated with engaging in NSSI for automatic/intrapersonal reasons. Similarly, Yates and colleagues [12] also found that individuals with recurrent NSSI were more likely to endorse intrapersonal reasons.

In the final models, gender was significantly associated with autonomic functions, but not with social functions. Previous studies have also found that females were more likely to engage in NSSI for intrapersonal functions [32], and perhaps especially to punish oneself [2,35,57], whilst there was no gender difference with regard to social functions [32].

After controlling for frequency of NSSI and gender, emotional abuse was a significant predictor in the automatic model, but not in the social model, indicating that there is a unique relationship between self-reported experiences of emotional abuse and the functions that mainly refer to emotion regulation. Even after the mediating effects of symptoms of depression and dissociation were controlled for, the relationship was still significant. Previous research has found a relationship between NSSI and emotional abuse [9-11] and interest has been directed toward the effect of invalidating environments and emotional dysregulation (e.g., [30]). The results in this study contribute further support for the hypothesis that emotional abuse results in intrapersonal vulnerabilities with difficulties in regulating emotions [1], where one strategy to regulate emotions could be to engage in NSSI.

Self-reported handicap or illness of a chronic nature was significant in the model of automatic functions. Such vulnerabilities may make an individual more exposed to adversities and influence the capacity to use alternative approaches when experiencing distress, perhaps instead turning to NSSI to regulate emotions. There is however a lack of information as to what kind of handicap or illness this variable refers to.

Symptoms of depression uniquely predicted automatic/intrapersonal functions but not social functions, corroborating previous findings that symptoms of depression are involved in the emotion-regulating functions of NSSI [32,34-36]. Further support for the role of depression in NSSI has been found in longitudinal studies [23-25].

### Automatic and social functions of NSSI

Physical abuse was a significant predictor in both models. There has previously been some inconsistency as to which kind of child maltreatment is related to NSSI. A recent study of 11,423 Australian adults, examining the relationship between child maltreatment and NSSI, showed a particularly strong association for physical child abuse and subsequent NSSI [7]. Other studies have also found support for such a relationship [5,6,9]. In one study of 58 psychiatric outpatients, physical abuse rather than other kinds of childhood maltreatment was significantly related to self-injury [8]. Muehlenkamp et al. [58] showed that those with repetitive NSSI were more likely to have experienced physical abuse. Their results support the detrimental effect of physical abuse and they further showed that those with self-injury who had also experienced physical abuse had more self-reported difficulties in identifying, recognizing, and being aware of emotional experiences. In this context, Yates [31] has argued that experience of physical abuse can lead to detachment from the body and a probable desensitization to physical pain, which might be one explanation why individuals with such experiences turn to NSSI rather than to less painful experiences [58]. In this context, it is also possible to understand the association between physical abuse and dissociation found in both automatic and social functions.

Symptoms of dissociation were a significant predictor in both the automatic/intrapersonal and the social/interpersonal model. Dissociation was also a significant mediator between different abuse types (emotional, physical, sexual) and automatic functions, as well as mediating the relationship between physical abuse and social functions. The role of dissociation in NSSI has previously been discussed with regard to the mechanism of why individuals engage in NSSI (e.g., [9,13]). Gratz et al. [4] found dissociation to predict self-harm in their multiple regression model for both men and women.

A previous suicide attempt was a significant predictor in both the automatic and social models, such as engaging in NSSI in order to escape unbearable emotional experiences and also to communicate with others. That suicide attempts also predicted social functions was contrary to our hypothesis, inconsistent with results by Nock and Prinstein [36], but in line with the study from Klonsky and Glenn [32]. Those with both NSSI and suicide attempt have previously been found to be a specially burdened and distressed group [22]. These results further underscore that socially reinforced NSSI is not synonymous with the absence of psychopathology [36].

Reports of physical abuse, suicide attempt and symptoms of dissociation were thus all significant predictors in the final models of both automatic and social functions. These results can tentatively be seen in the light of Joiner’s theory of suicide [59], which postulates that repeated painful experiences (such as physical abuse) together with past self-injury may habituate an individual to pain and provocation, potentially leading to the ability to cause lethal self-injurious behavior in the context of thwarted belongingness and being perceived as a burden, increasing the need to regulate both emotional and social experiences. This is also consistent with the findings by Baetens et al. [60], who found that adolescents with suicidal self-injury reported more stressful life events and more physical abuse than adolescents with non-suicidal self-injury.

### Social functions of NSSI

Symptoms of anxiety was the only predictor that uniquely predicted social, but not automatic functions. Klonsky and Glenn [32] also found that anxiety was associated with the social functions of NSSI. Perhaps anxiety represents an affective state, which is “characterized by more elevated arousal or heightened approach motivation” [44, p. 4], compared to depression, for example, which could explain its relationship with engaging in NSSI to communicate with and try to influence others.

### Non-significant variables

Contrary to our hypothesis, when frequency of NSSI was included in the model and controlled for in the analyses, sexual abuse was not a significant predictor when studied together with other maltreatment variables and trauma symptoms. When frequency of NSSI was not included in the model, there was a non-significant trend for sexual abuse in the model of automatic, but not social functions, indicating that it is possibly more closely related to NSSI functions of emotion regulation, feeling-generation and self-punishment. Further analyses showed that frequency of NSSI mediated the relationship between sexual abuse and automatic functions. Kumar et al. [35] have previously found support for a relationship between sexual abuse and a punitive function of NSSI, and in a study by Kaess et al. [37] there was a unique relationship between sexual abuse and automatic, but not social functions. Some studies have found support for a relationship between sexual abuse and NSSI [5,11,12], and results by Klonsky and Moyer [14] showed that the association was stronger for clinical samples than for normative samples.

The variable referring to parental chronic adversities was not significant in either model, suggesting that directly experienced abuse toward the adolescent (e.g., emotional and physical abuse) during upbringing is a stronger predictor of the automatic and social functions of NSSI than more indirect adversities that were measured with the parental chronic adversities variable. This variable, however, did not contain information about the quality of the parent–child relationship, despite the presence of parental alcoholism, for example, or mental health problems etc. Variables of criticism, hostility and alienation were not measured in this study, and it might be that the family environment was not detrimental or invalidating despite one or both parents struggling with mental illness or alcoholism, for example.

Contrary to our hypothesis, symptoms of posttraumatic stress were not a significant predictor in the model of automatic functions of NSSI. This is inconsistent with previous results, where symptoms of posttraumatic stress were associated with the feeling-generation function [36], and have also been shown to be a mediator between maltreatment and self-injury [27,28]. Other studies (e.g., [27,36]), however, used a comprehensive interview to assess posttraumatic stress, which might explain the discrepancy of results compared to the subscale of self-reported symptoms of posttraumatic stress from the TSCC used in this study.

Inconsistent with previous results from Hilt et al. [34], and contrary to our hypothesis, peer victimization/bullying was not a significant predictor in the model of social functions, although it has recently been shown to be a factor to take into consideration in the understanding of NSSI [61]. One explanation for this could be the use of a general single-item assessment in this study, which usually generates less valid results. The relationship between social functions and bullying thus needs to be examined further.

### Study strengths and limitations

This study used a large community sample of self-injuring adolescents. As pointed out by Klonsky and Glenn [32], a non-clinical sample of self-injurers includes participants both with and without serious psychopathology, which is a strength when it comes to generalization. Social/interpersonal functions have previously been shown to be a more prevalent function in community samples of adolescents [2], compared to clinical groups [36]. It is therefore important to also include non-clinical samples when examining different functions of NSSI. A further strength in the present study is that well-established self-report measures were used. This is especially important in the research field of NSSI, which has been plagued by different definitions and confusion concerning terminology. Another strength is that the study measured several different types of maltreatment and adversities that were tested together in multiple regression models.

Some limitations, however, need mentioning. The 272 excluded adolescents did not differ from those included on background demographics, but there were significant differences in self-injury status. Those excluded due to too many missing items on the functions of NSSI reported less suicide attempts, less frequent NSSI as well as less moderate/severe NSSI as defined by Lloyd et al. [44]. In all likelihood this meant that those with non-significant NSSI were excluded. Perhaps they had only tried a minor NSSI once or twice and didn’t consider it necessary to fill in the functions. This interpretation is supported by Lloyd-Richardson et al. [2], who showed that minor injurers were more likely to deny engaging in NSSI for any of the reasons listed in FASM, as compared to moderate/severe injurers. Although the present study found support for mediation, it is necessary to point out that results only support statistical mediation. Since the study design was cross-sectional, the directions of relationships cannot be determined, precluding conclusions regarding causality. Further, the questions about NSSI and functions referred to the last year, whereas the questions about trauma symptoms referred to a time period of the last two weeks. It is, for example, possible that NSSI may lead to increased depressive symptoms. The evidence here is contradictory; although one longitudinal study [24] found evidence for a bidirectional relationship in girls, with depressive symptoms being a risk factor for increased self-harm one year later and self-harm a risk factor for increased depressive symptoms one year later. Another longitudinal study [25] found depression to be a predictor of NSSI and not the other way around. Retrospective self-report is known to involve error due to forgetting and biased recall. Another limitation is that there was single-item assessment of maltreatment/abuse experiences, despite the fact that experiences such as these are seldom clear-cut and categorical [8]. There is a lack of data on general psychopathology and differential diagnoses in this sample. There is therefore no way of discriminating between adolescents with NSSI with or without symptoms of borderline personality disorder (BPD), for example, which is a limitation since symptoms of BPD can potentially influence the reported functions of NSSI v[32,62]. Furthermore, symptoms of depression, anxiety, anger, posttraumatic stress and dissociation were only assessed as subscales of the TSCC and not as validated diagnoses or as traits.

### Clinical implications

Adolescents engage in NSSI for a reason, and in order for them to be able to replace NSSI with alternative (functionally equivalent) behaviors, it is important to understand the specific context in which NSSI has developed and is maintained. In order to understand the mechanisms underlying the need to engage in NSSI to regulate emotional and/or social experiences it is not only necessary to assess experiences of negative life events but also more proximal individual psychopathology, such as level of trauma symptoms, since they can have a mediating effect on the complex relationship. An assessment of the specific reinforcing functions of NSSI can be helpful to guide functionally relevant individualized treatment strategies. In this study, experiences of physical abuse, a previous suicide attempt and symptoms of dissociation were found to be involved in the pathway to engaging in NSSI for regulating both emotional and social experiences. These factors can thus be indicative of a broad treatment approach aimed at emotion regulation skills as well as interpersonal skills in the context of the caregiving environment, in which it is equally important to attempt to reduce adversities, conflict and criticism and encourage validation and pay attention to low-key attempts of social signaling. Furthermore, experiences of emotional abuse and symptoms of depression in adolescents with NSSI can potentially alert clinicians to interventions aimed at helping the adolescent and caregivers with emotion regulation skills, in order to reduce the need for NSSI to regulate emotions, generate feelings and to self-punish.

## Conclusions

To summarize, this study contributes important knowledge of which individual proximal trauma symptoms are involved in the process through which distal adverse environmental events might influence engaging in NSSI for either automatic or social functions. The study found that NSSI frequency, gender (female), emotional and physical abuse, prolonged illness or handicap and symptoms of depression and dissociation were all significant predictors in the final model of automatic functions, indicating that these variables are important in understanding the mechanism behind the need to engage in NSSI to regulate emotions, generate feelings or to self-punish. Symptoms of depression and dissociation mediated the relationship between maltreatment and automatic functions. Frequency of NSSI, gender, emotional abuse, prolonged illness or handicap and symptoms of depression uniquely predicted automatic functions but not social functions. Physical abuse, having made a suicide attempt, symptoms of anxiety and dissociation were significant in the final social model. Of these, symptoms of anxiety were uniquely associated with social, but not automatic functions. Symptoms of anxiety and dissociation also mediated the relationship between physical abuse and performing NSSI for social functions. Although both models were significant, the predictors explained more variance in the automatic functions compared to the social functions, indicating that the predicting variables included in the model are meaningful constructs in relationship to the automatic functions of NSSI. Social functions are to a larger degree explained by additional variables not included in the present study. These models have potentially important implications for developing long-needed functionally relevant treatment strategies for NSSI.

## Competing interests

The authors declare that they have no competing interests.

## Authors’ contributions

MZ conceived the study, participated in the design of the study, performed the data collection and the statistical analysis and helped draft the manuscript; L-GL helped draft the manuscript and revised it critically; CGS participated in the design of the study, helped draft the manuscript and revised it critically. All authors read and approved the final manuscript.
